# Cultured Human Thymic-Derived Cells Display Medullary Thymic Epithelial Cell Phenotype and Functionality

**DOI:** 10.3389/fimmu.2018.01663

**Published:** 2018-07-23

**Authors:** José A. Villegas, Angeline Gradolatto, Frédérique Truffault, Régine Roussin, Sonia Berrih-Aknin, Rozen Le Panse, Nadine Dragin

**Affiliations:** ^1^INSERM, AIM, Center of Research in Myology, UMRS974, Sorbonne University, Paris, France; ^2^Hospital Marie Lannelongue, Le Plessis-Robinson, France; ^3^Inovarion, Paris, France

**Keywords:** thymic epithelial cells, primary cell culture method, keratins, cytokines, chemokines, tissue-specific antigens

## Abstract

Thymic epithelial cells are one of the main components of the thymic microenvironment required for T-cell development. In this work, we describe an efficient method free of enzymatic and Facs-sorted methods to culture human medullary thymic epithelial cells without affecting the cell phenotypic, physiologic and functional features. Human medulla thymic epithelial cells (mTECs) are obtained by culturing thymic biopsies explants. After 7 days of primo-culture, mTECs keep their ability to express key molecules involved in immune tolerance processes such as autoimmune regulator, tissue-specific antigens, chemokines, and cytokines. In addition, the cells sensor their cultured environment and consequently adjust their gene expression network. Therefore, we describe and provide a human mTEC model that may be used to test the effect of various molecules on thymic epithelial cell homeostasis and physiology. This method should allow the investigations of the specificities and the knowledge of human mTECs in normal or pathological conditions and therefore discontinue the extrapolations done on the murine models.

## Introduction

The thymus is a primary lymphoid organ where T cell development and maturation take place. The thymic stromal cells correspond to heterogeneous cell types including mainly thymic epithelial cells (TECs) but also dendritic cells (DCs), macrophages, myoid cells, and fibroblasts. They create a tridimensional network to establish close contact with T-cells to support and to direct T-cell differentiation, maturation, and selection (1–3).

Derived from a common progenitor (4–6), two types of TECs based on their localization in the thymus are defined: medulla thymic epithelial cells (mTECs) and cortical thymic epithelial cells (cTECs) (7). cTECs contribute to the T cell lineage commitment and the positive selection, processes that allow the development of T cells exhibiting specific T cell receptor with low or high affinity to MHC molecules (8). mTECs complete and finalize T cell maturation processes through the negative selection of T cells as well as the generation of regulatory T cells (Tregs) and natural killer T cells (NKT) (9–11). To ensure their role in immune tolerance, mTECs express a wide range of proteins that correspond to self-antigens expressed by peripheral tissues and organs. The expression of these tissue-specific antigens (TSAs) in mTECs is regulated by specific transcription factors: autoimmune regulator (AIRE), Fez family zinc finger protein 2 (FEZF2), and PR domain zinc finger protein 1 (Prdm1) (12, 13). As a consequence, in mice with mutations in these genes, mTECs are defective in their tolerance process and mice are associated with autoimmune phenotypes. In addition, mTECs express various cytokines [interleukin 6 (IL-6), interleukin 1β (IL-1β)], and chemokines (CCL19 and CCL21) (14–17) that are critical for the T cell signaling and migration throughout the cortex/medulla compartment. Therefore, alterations in mTECs are deleterious for T cell differentiation and can lead to complete blockage of thymopoiesis (18) or reduce thymocyte proliferation (19), as illustrated in thymuses of knockout mice for FOXN1, and CBX4. Foxn1 is a transcription factor that regulates, during the thymus development, the expression of CCL25, Dll4, and Hoxa3, factors required for the thymocyte and TEC differentiation (20). Furthermore, CBX4 is a crucial and non-redundant protein that controls the generation and maintenance of the thymic epithelium (19).

The functional role of TECs has been studied using well-established mouse models (21–23) while few human models are available (24–29). These numerous models reveal murine mTEC features and functions, but they cannot apply to human mTEC specificities. Indeed significant steps are characterized by dissimilar kinetic expression of specific markers in humans versus mice (30). So far, few models are developed in humans. Fernandez et al. have characterized cloned TEC lines derived from human cortical epithelium (31). In addition, Patel et al. have found that TECs obtained from cultured human explants (32), display similarities to keratinocytes compared with others cell types by screening membrane markers. An enzymatic procedure to isolate and then to culture human primary TECs has been set up (33) and has later been characterized by Skogberg et al. (34). However, it is reported that enzymatic digestion can affect expression level of surface molecules (35) and then influence the purification process and the cell viability (36). To avoid these issues, here we describe an efficient enzyme-free procedure to culture primary human mTECs by using fresh thymic explants. This method allows in a reduced processing time and with a limited tissue amount, to obtain a significant number of cells displaying mTEC features and expressing specific TEC surface markers and TSAs. This model is a viable cell model indispensable to investigate human mTEC physiology in normal or pathological conditions.

## Materials and Methods

### Human Samples

Human thymic fragments (5–10 g) were obtained from immunologically normal male and female newborns (age 2 days to 1-year old) undergoing corrective cardiovascular surgery at the Marie Lannelongue Surgical Center (Le Plessis-Robinson, France). To perform RNA and protein analyses, tissue samples were fast frozen in liquid nitrogen. To establish primary human thymic epithelial cells (TECs), pieces of tissue were placed in sterilized RPMI medium and maintained at 4°C until cell culture procedure.

This study was under the French Bioethic Law that requires a written informed consent from the donors or the legal representant. In respect to this law, this study was approved by the local ethics committee (CPP, Kremlin-Bicêtre, France: agreement No. 06-018; CCP Ile de France Paris 7, France agreement No. C09-36).

### Primary Cell Cultures

Primary human TEC cultures were established on the basis of previous reports (11, 37, 38). Human thymic tissues were cut with scissor into small fragments (~10 mm^2^) in Hank’s balanced salt solution (HBSS) medium and then washed three to four times in HBSS medium to discard most of thymocytes. The washed thymic explants were transferred to 75-cm^2^ culture dishes and were allowed to attach to the flask for 5 min without medium. Ten ml of fresh culture medium were added gently and flasks were placed in a chamber incubator at 37°C with 5% CO_2_. The culture medium was prepared with RPMI 1640 supplemented with 20% horse serum (Life Technologies, Cergy-Pontoise, France), 0.2% Ultroser G (Biosepra, Cergy, France), 2 mmol/l of l-glutamine, 100 IU/ml of penicillin, and 100 µg/ml of streptomycin (Life Technologies, Cergy-Pontoise, France). Twice a week, the medium was removed, the cells were washed once with phosphate-buffered saline (PBS) 1× (Life Technologies, Cergy-Pontoise, France), and a fresh culture medium was added. Rapidly, cells migrated out of the thymic explants and expanded around the biopsies. After 7–8 days, cells were washed with PBS, and a first trypsin treatment [0.075% trypsin (Life Technologies, Cergy-Pontoise, France) and 0.16% EDTA (Life Technologies, Cergy-Pontoise, France)] was performed during 3 min to remove fibroblasts that could have grown in the cultures. Primary human TECs remained attached to the flasks, and then they were rinsed with PBS. A new trypsin solution was added, and TECs were collected by adding 5 ml of 0.075% trypsin solution for 5 min at 37°C. The content of each flask including detached cells and explants was filtered using 40 µm cell strainer (Dutscher, Brumath, France). Then, the explants were removed, and the individual cells were diluted in culture medium devoid of Ultoser-G and supplemented with 5% horse serum. The nature of TECs and the percentage of medullary TECs were analyzed as described below before being seeded at a density of 2–3 × 10^3^ cells into 12 or 24-well Nunc cell plates (Life Technologies, Cergy-Pontoise, France). Cells were allowed to attach to the plates for 24 h before being treated for 24 h.

Thymocytes were isolated from human thymi by mechanical dissociation of fresh thymic tissue, as previously described (39, 40). The cells were filtered through cell strainer advice to remove thymic tissues and washed once with HBSS. Thymocytes were quick frozen upon RNA extraction.

Fibroblasts were obtained after 7 days of thymic explants culture during the first trypsin step as previously described by Cufi et al. (41).

### Immunofluorescence Microscopy

Cryostat sections (7 µm) of frozen thymic tissues and primary cultured TECs were fixed with acetone to glass superfrost slides and lab-teck chamber slides, respectively, and dried for 1 h. The thymic sections and primary cultured TECs were pre-incubated with a blocking buffer (PBS 1×, 0.1% bovine serum albumin, 10% fetal bovine serum, 0.3 M glycine, and 1% Tween) for 1 h at room temperature and, then incubated for 2 h at room temperature with antibodies raised against human antigens listed in Table S1 in Supplementary Material. The labeled cells were revealed with Alexa 488- and/or Alexa 594-coupled secondary IgG raised in donkey or chicken, respectively. Images were acquired with a Zeiss Axio Observer Z1 Inverted Microscope using 20× magnification (Carl Zeiss, Le Pecq, France).

At day 7 of culture, the nature of TECs and the percentage of medullary TECs were estimated by flow cytometry and immunofluorescence using MNF116 antibody (Dako, Trappes, France) and an anti-keratin 5/14 antibody (Covance, Rueil-Malmaison, France) (42, 43), respectively. The MNF116 antibody is a pan-cytokeratin antibody that recognizes various cytokeratins 5, 6, 8, 17, and 19 (K5, K6, K8, K17, and K19). The anti-keratin 5/14 antibody recognizes the medullary TECs on thymic sections (44). An anti-collagen III antibody (Clone III-53; ICN) was used to identify fibroblast cells. Under a percentage of 80% of TEC cells, cells were discarded because the presence of other contaminating cells, mainly fibroblasts (Figures S1 and S2 in Supplementary Material).

### RNA Extraction

Total RNA was prepared from the thymus and TECs using the trizol RNA Isolation kit (Life Technologies, Cergy-Pontoise, France). Thymuses were homogenized with the FastPrep FP120 instrument (Qbiogen, Illkirch, France). The concentration of RNA was analyzed with a NanoDrop ND-1000 spectrophotometer (LabTech, Palaiseau, France). RNA samples presenting a minimal ratio of 1.9 and 2 for, respectively, 260/280 and 260/230 were also controlled on a gel. The RNA quality was assessed with the ARN FlashGel™ System. When the samples were degraded even partially, they were excluded.

### Real-Time PCR

The reverse transcription of total RNA into cDNA was performed by using the SuperScript II RT kit (Life Technologies, Cergy-Pontoise, France) according to the manufacturer’s instructions. PCR reactions were completed using the LightCycler apparatus as previously described (38). The primers used for the real-time PCR are listed in Table S2 in Supplementary Material. Each cDNA sample was run at least in duplicate.

### Flow Cytometry

Flow cytometry analysis of the MNF116 expression in primary cultured TECs was done according to the protocol described by the manufacturer. MNF116 antibody recognizes cytokeratins (5, 6, 8, 17, and 19). Cells were fixed with 4% paraformaldehyde and then permeabilized with 0.1% PBS-Tween. The permeabilized cells were incubated with either an uncoupled mouse anti-MNF116 antibody (Dako, Trappes, France) or uncoupled mouse anti-collagen III antibody (ICN Biomed, Illkirch, France) and washed twice with PBS 1×. MNF-116 or collagen III staining was detected with Alexa 488 coupled chicken anti-mouse IgG (Life Technologies, Cergy-Pontoise, France). After two washings with PBS, cells were analyzed on the FACSVerse apparatus using the Facs suite software (Becton Dickinson, Le Pont-de-Claix, France).

### Statistical Analysis

ANOVA and Mann–Whitney tests were used as specified in each figure. Statistical significance is recognized at *p* < 0.05. GraphPad prism was used for all the statistical analysis.

## Results

### Human Thymic-Derived Cells Display mTEC Morphology and Surface Markers

**Figure 1:**
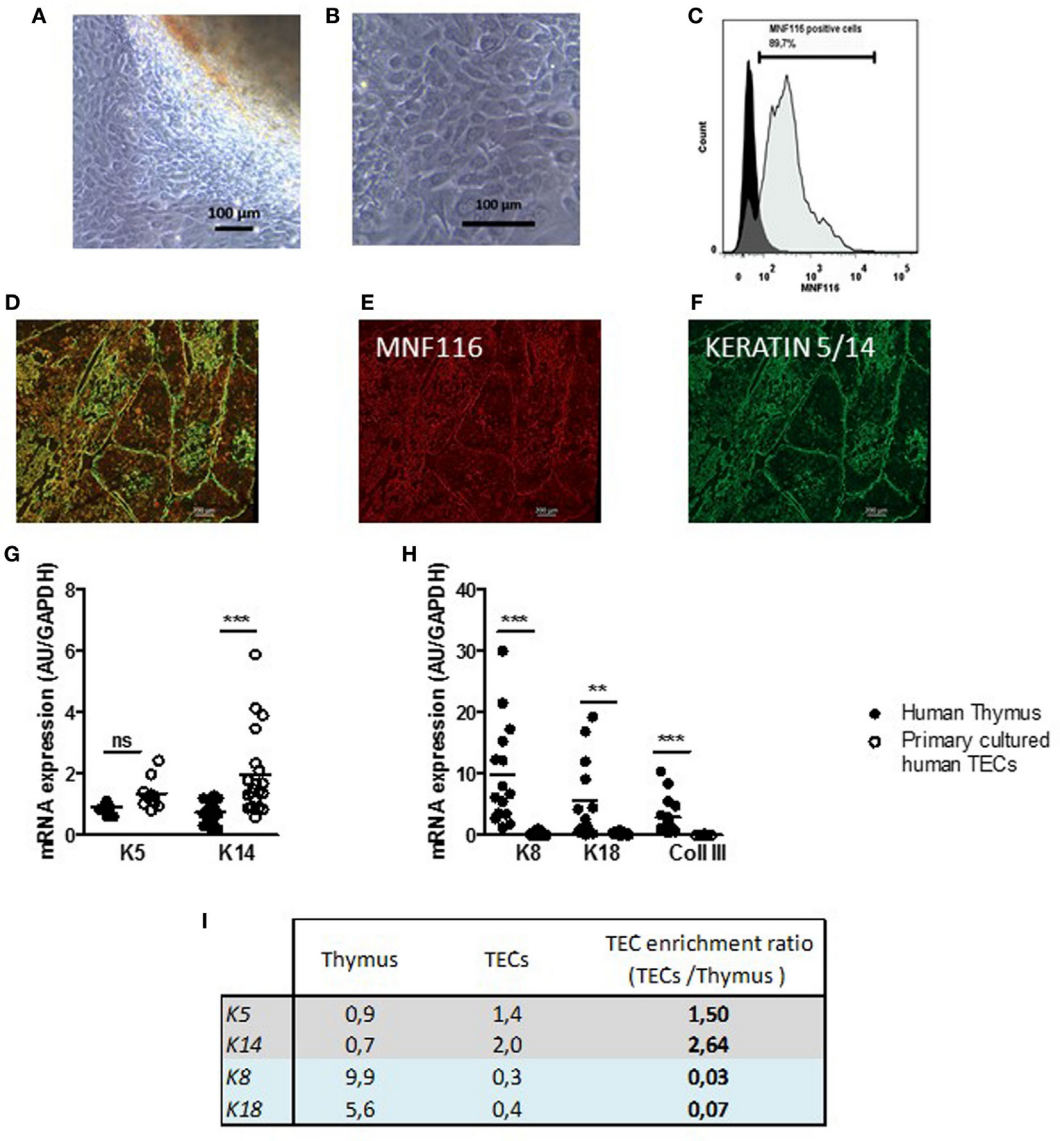
Primary cultured human thymic stromal cells display thymic epithelial cell (TEC) features. **(A,B)** Representative pictures of thymic cells expanding from human thymic explants. **(A)** After 2–4 days of culture, cells migrate and proliferate out of the explants in culture. **(B)** Thymic cell culture with the morphologic mosaic form of epithelial cells. **(C)** Representative flow cytometry plot of primary cultured TECs labeled with an anti MNF-116 antibody; the control isotype is shown in black at a day 7. **(D)**. Representative pictures of primary human thymic sections **(E–G)** co-labeled with an anti-MNF-116 antibody (red) **(E)**, and anti-keratin 5 and 14 antibodies (green) **(F)**. Images were acquired with a Zeiss Axio Observer Z1 inverted microscope. mRNA expression levels of medulla thymic epithelial cell (mTEC) markers (*K5* and *K14*) **(G)**, cortical thymic epithelial cell (cTEC) markers (*K8* and *K18*), and a fibroblast cell marker (collagen III, *Coll III*) **(H)** in human thymuses and in primary cultured TECs (day 6–7). Enrichment ratios of gene expression of specific markers for mTECs (gray) and cTECs (blue) in cultured human TECs (day 7) versus total thymus **(I)**. mRNAs were analyzed by real-time PCR and normalized to GAPDH for total thymuses and primary TECs. mRNAs are expressed as arbitrary units and are the mean values (*n* > 9 for thymuses and *n* > 5 for different donors for primary cultured TECs). *p* Values were obtained using the ANOVA test. Asterisks indicate significant differences (**p < 0.005 and ***p < 0.0001).

Cultures of human thymic explants could lead to heterogenous cultures of thymic cells. However, our culture conditions mainly allowed the proliferation and survival of thymic stromal cells, since non-adherent cells namely the thymocytes remain in the supernatants and are eliminated during washing steps. After 2–4 days, the primary thymic cells expanding from the thymic explants (Figure 1A) displayed closely packed cells that harbored the morphologic mosaic form of epithelial cells (Figure 1B). The cultured cells stained positively to MNF-116 (Figure 1C), a pan-cytokeratin antibody that recognizes various cytokeratins 5, 6, 8, 17, and 19 (K5, K6, K8, K17, and K19) expressed in thymic medullary and cortical epithelial cells. As observed in Figures 1D,E on thymic sections, the MNF116 antibody labeled the entire thymic epithelial network (medulla and cortical areas), while the anti-K5/K14 antibody stained specifically the thymic medulla area (Figure 1F). These data suggested that whether primary cultured thymic cells display morphological and phenotypical appearance of TECs using the MNF116 antibody did not discriminate cTECs from mTECs in thymic-derived cell cultures. Nevertheless, inherent to the thymic explants, cell populations other than TECs could be found in low proportion as shown in Figure S1 in Supplementary Material. At day 7–8, when fibroblast cells considered as contaminants, exceeded 20%, the culture was discarded. Fibroblasts were identified by staining with anti-collagen 3 antibody (Figure S2 in Supplementary Material).

To further characterize TECs, at day 7 of culture, we quantified by qPCR the level of expression of various TEC markers in thymic biopsies and the primary cultured TECs (Figures 1G,H). We showed that thymic biopsies expressed mainly cortical (*K8/K18*) and fibroblast markers (Figure 1H). By contrast, primary human TECs expressed mainly medullary markers (*K5* and *K14*) (Figure 1G); cortical keratins (*K8* and *K18*) and fibroblastic marker (*COLLAGEN III*) expressions were barely detectable (Figure 1G). By using the microdissection technology on human thymus sections, we showed that 82% of *K5* thymic expression and 87% of *K14* thymic expression were due to the medullary microdissected areas of human thymuses while K8 was mainly cortical (Figure S3 in Supplementary Material). The compared analysis of the gene expressions and their ratios in TEC cultures versus thymic biopsies, confirmed that our culture method sustained the growth of cells expressing predominantly medullary markers such as *K5* and *K14*, with an enrichment ratio between 1.5 and 2.64 while it was below 0.03 for cortical markers (Figure 1I).

Medulla thymic epithelial cells are a heterogeneous cells composed of different cell subpopulation identified by functional, phenotypic, and developmental markers (45–47). We wondered whether this diversity is maintained in culture. To this end, we investigated the protein expression of various specific mTEC markers, such as CLAUDIN 4, CLAUDIN 3, tight junction components known to be expressed by mTEC lineage-committed cells and *Ulex europaeus* agglutinin-1 (UEA) lectin (27, 48, 49), a marker of highly proliferative mTECs expressing autoimmune regulator (AIRE) protein (45).

**Figure 2:**
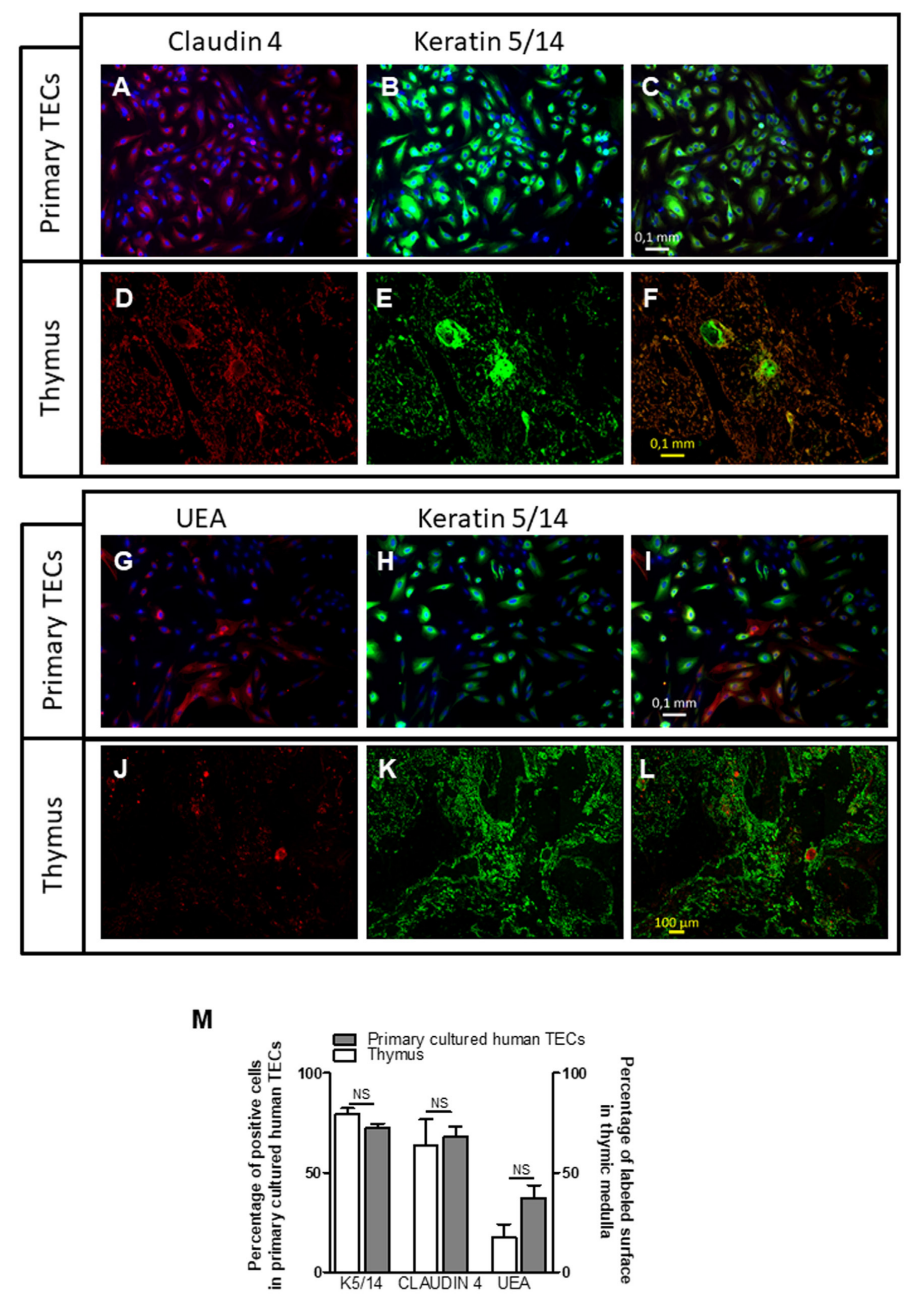
Primary cultured human thymic cells display medulla thymic epithelial cell features. Representative pictures of a primary cultured human thymic epithelial cells (TECs) (day 7) **(A–C)** and human thymus **(D–F)** co-labeled with an anti-Claudin 4 antibody (red), anti-keratin 5, and 14 antibodies (green). Representative pictures of primary cultured human TECs **(G–I)** and human thymus **(J–L)** co-labeled with an *Ulex europaeus* agglutinin I lectin (UEA) (red), anti-keratin 5 and 14 antibodies (green). The percentage of positive cells in primary cultured human TECs represented the number of KERATIN 5/14, CLAUDIN 4, or UEA positive cells versus the total cell number **(M)**. For thymic sections, the surface of KERATIN 5/14 or CLAUDIN 4 positive areas was measured and compared with the thymic medulla. Images were acquired with a Zeiss Axio Observer Z1 Inverted Microscope using 20× magnification. The counting was done as previously described in Dragin et al. (50). ImageJ software was used to display the digital pictures and to count manually the labeled cells. Graph bar represents the results obtained with four different human biopsies and primary cultured human TECs. The non-parametric Mann–Whitney test was used for statistical analyses.

Figure 2 showed that cultured cells exhibited positive labeling for K5/14, for CLAUDIN 4 (Figures 2A–C) as compared with thymic biopsies (Figures 2D–F). These labeling mirrored the medulla compartment of the thymus tissue (Figures 2D–F). The UEA antibody labeled few cultured mTECs (Figures 2G–I). Similarly, few mTECs in human thymic sections were stained with this antibody (Figures 2J–L). The percentage of positive cells in cultured mTECs and in the thymic medullary areas is shown for the different markers in Figure 2M, and no statistical differences were observed. Altogether, these data showed that our culture model maintained a diversity of the mTEC subpopulations comparable with that in global thymuses.

### Human Primary Cultured mTECs Express Factors Involved in T Cell Negative Selection Process

**Figure 3:**
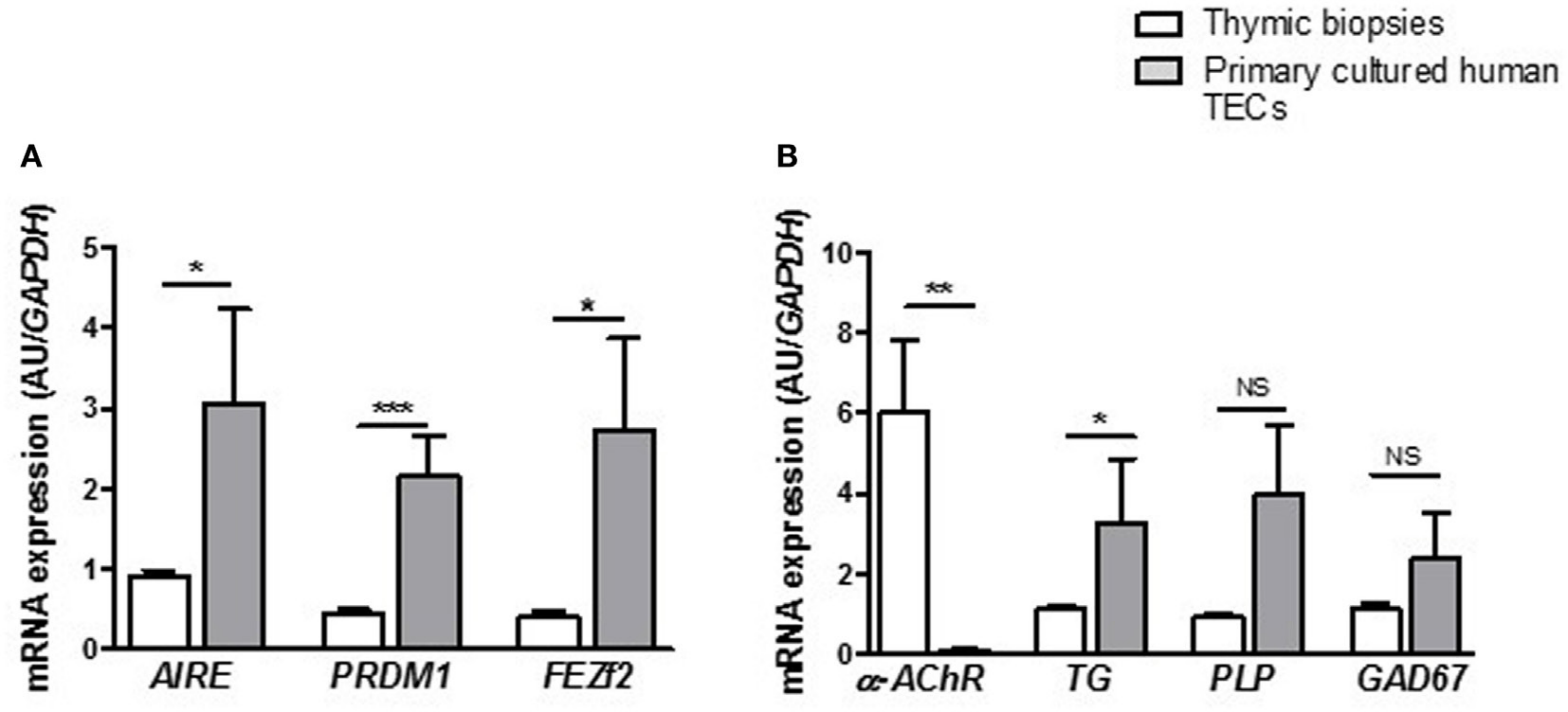
Primary human thymic epithelial cells (TECs) express immune tolerance molecules. mRNA expression levels of transcription factors **(A)** and tissue-specific antigens **(B)** in human thymuses and in primary cultured TECs. mRNA expressions were analyzed in primary cultured TECs at day 7 of culture. mRNAs were analyzed by real-time PCR, normalized to *GAPDH*, and are expressed as arbitrary units (±SEM) (*n* > 5 for thymuses and *n* > 5 different donors for primary cultured TECs). *p* Values were obtained using the non-parametric Mann–Whitney test. Asterisks indicate significant differences (*p < 0.05, **p < 0.007, and ***p < 0.0001).

Medulla thymic epithelial cells play a major role in immune tolerance by expressing and presenting TSAs to developing T cells. TSAs expression in mTECs is controlled by various transcription factors among them AIRE, FEZf2, and PRDM1. We evaluated the ability of cultured primary TECs to express such tolerance markers. At day 7, we observed that primary cultured TECs expressed *AIRE, PRDM1*, and *FEZF2* (Figure 3A) and various TSAs, such as the α-acetylcholine receptor (α*-AChR*), thyroglobulin (*TG*), myelin proteolipid protein (*PLP*), and glutamic acid decarboxylase 67 (*GAD67*) (Figure 3B). The significant high expression of these various factors in primary cultured TECs compared with the thymic tissue highlighted that analysis of gene expression in global tissue (which is a mix of various cell types) may underestimate the gene expression in a cell type. More, these data suggested that cultured mTECs are probably functional since they remain able to express these specific functional markers. For α-AChR, the gene expression ratio between cultured TECs and total thymus did not display an increase. This is in part due to the fact that in the thymus, the AChR subunits are highly expressed in myoid cells (muscle-like cells) compared the TECs (51).

### Human Primary Cultured TEC Express Cytokines and Chemokines

**Figure 4:**
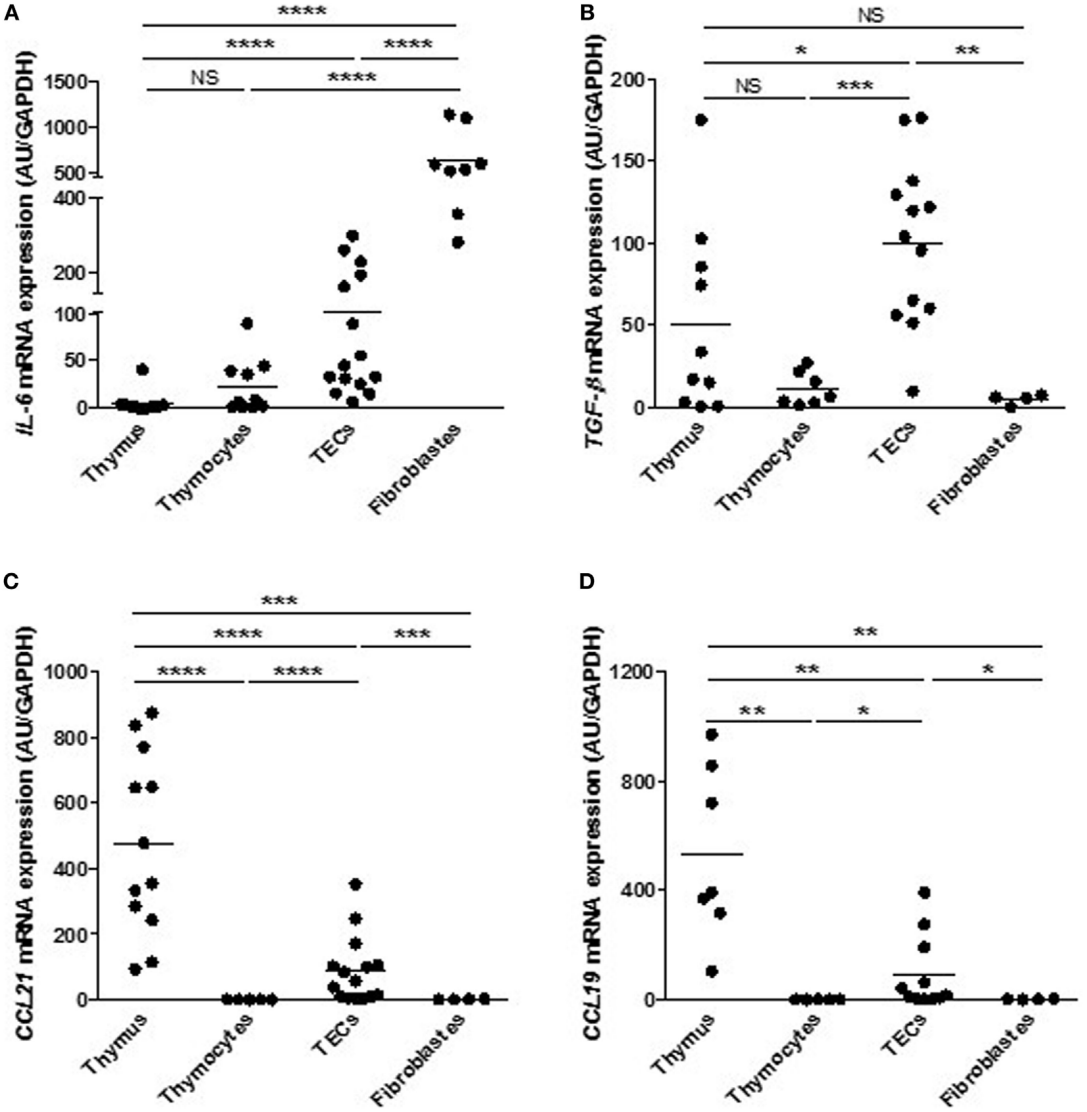
Primary cultured human thymic epithelial cells (TECs) express cytokines and chemokines. Comparison of mRNA levels of interleukin 6 (*IL-6*) **(A)**, tumor growth factor-β (*TGF-β*) **(B)**, *CCL21* **(C)**, and *CCL19* **(D)** in different thymic cell types. mRNAs were analyzed by real-time PCR and normalized to *GAPDH*. For each gene, mRNA expressions in primary TECs were normalized to 100 and compared with others cell types. mRNA expression levels are expressed as arbitrary units and are the mean values (*n* > 6 for thymuses and *n* > 4 different donors for primary cultured TECs (day 7), fibroblasts or thymocytes). *p* Values were obtained using the Mann–Whitney test. Asterisks indicate significant differences (*p < 0.05, **p < 0.01, ***p < 0.001, and ****p < 0.0001).

Medulla thymic epithelial cell involvement in T cell development and maturation is not exclusively based on negative selection and tolerance process. To help T cells during their long journey across the thymus, mTECs secrete “guiding molecules” such as cytokines and chemokines that play a crucial role in T cell migration or signaling, and that could, in turn, affect TEC differentiation (52). We compared the expression level of cytokines and chemokines in our primary cultured TECs with other thymic cells (thymocytes and fibroblasts) and with thymic biopsies. We observed that cultured human primary TECs displayed a significant high mRNA expression of *IL-6* (Figure 4A), tumor growth factor-β (*TGF-β*) (Figure 4B), *CCL21* (Figure 4C), and *CCL19* (Figure 4D) compared with the other cell types. Of course, in human thymuses, different cell types may express *IL-6, TGF-β, CCL21*, and *CCL19*. However, in normal conditions, TECs appeared to be the main sources of TGF-β, CCL21 and CCL19 while IL-6 can also be provided by fibroblastic cells. Therefore, the data demonstrate that, after 7 days of culture, mTECs still displayed their ability to express molecules promoting their own differentiation as well as migration of thymocytes, validating the quality and the potential functional efficiency of the primary human mTEC culture model.

### Are Primary Cultured Human TECs Responsive to Stimuli?

It is well documented that estrogen targets mTEC transduction pathways, through its receptors (ER-α and ER-β) and then modulates the immune response (38, 53, 54). We have previously demonstrated that estrogen may modulate in mTECs, expression of AIRE and TSAs as well as cytokines and chemokines, such as IL-6 and CXCL13 (50, 55). More, the increased intrathymic level of inflammatory cytokines, such as IL-1α or IL-1β (27), during aging process, has been shown to affect TEC homeostasis and functionality (27). Consequently, primary TECs obtained from thymic explants were sub-cultivated and challenged with estrogen and IL-1β to evaluate their reactivity to stimuli.

**Figure 5:**
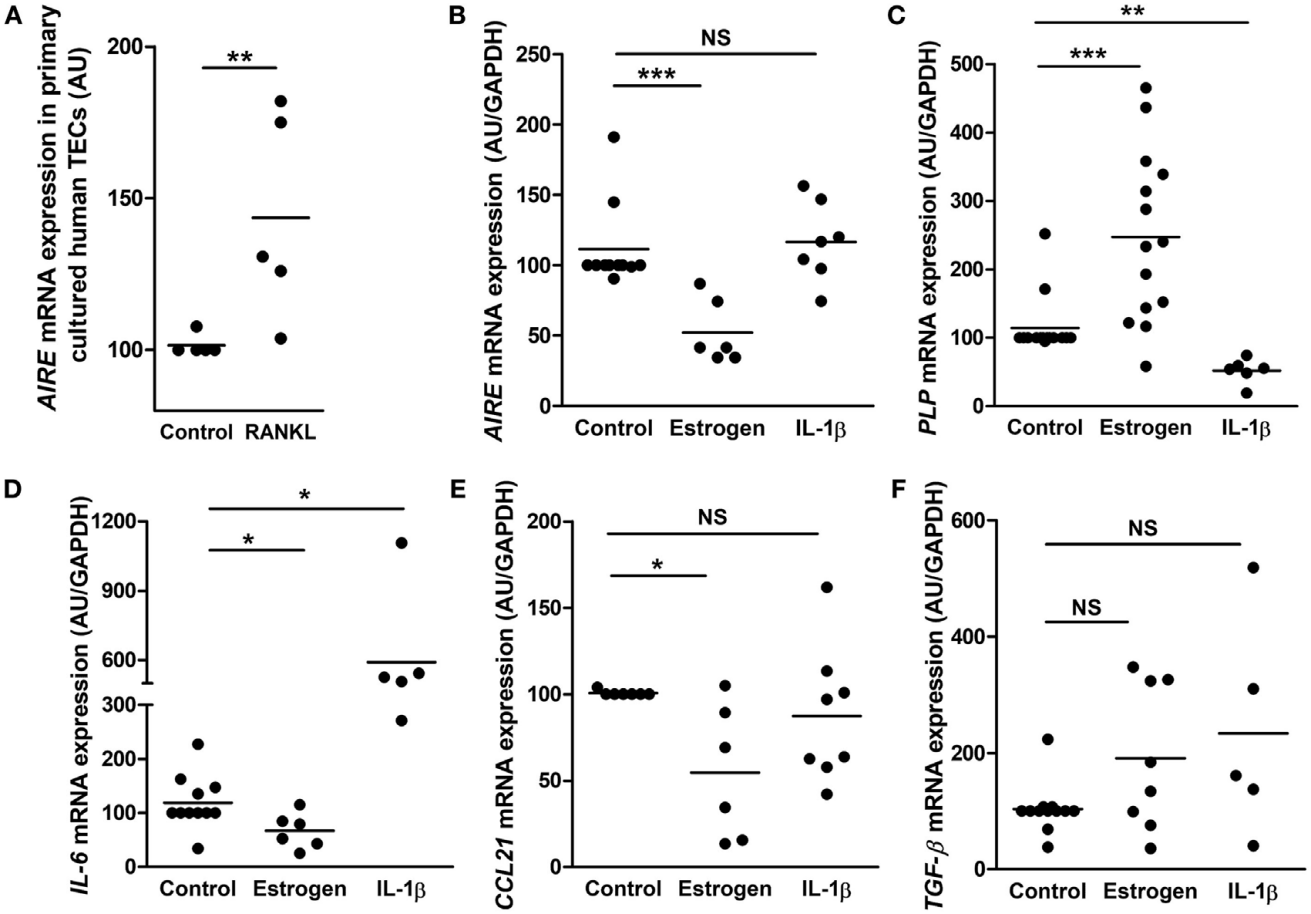
Effect of estrogen and interleukin 1β (IL-1β) on gene expression in primary cultured human thymic epithelial cells (TECs). Effect of RANKL (10^−9^ M) on *AIRE* mRNA expression **(A)**. Effect of estrogen (10^−8^ M) or IL-1β (1 ng/ml) on the mRNA expression of *AIRE* **(B)**, myelin proteolipid protein (*PLP*) **(C)**, interleukin 6 (*IL-6*) **(D)**, *CCL21* **(E)**, and tumor growth factor-β (*TGF-β*) **(F)** in primary cultured human TECs. For each experiment, cells were incubated for 24 h. mRNA expressions were normalized to 100 in control untreated cells. Each point represents the mean value of an experiment using primary cultured TECs (day 7) obtained from different donors (*n* > 5). mRNAs were analyzed by real-time PCR and normalized to *GAPDH*. *p* Values were obtained using the non-parametric Mann–Whitney test. Asterisks indicate significant differences (*p < 0.05, **p < 0.01, and ***p < 0.001).

First of all, we checked the gene expression induction capacity by the cultured mTECs. To this end, we added receptor activator of nuclear factor kappa-B ligand (RANKL) in the cell medium since *AIRE* mRNA expression is regulated by RANK/CD40 and lymphotoxin beta receptor signaling pathways (56–58). We observed a significant increase of AIRE mRNA expression (Figure 5A) suggesting that the cultured cells conserved their ability to overexpress AIRE upon stimulation.

Therefore, cultured primary human mTECs were challenged with estrogen and the inflammatory cytokine IL-1β to investigate the *in vitro* cell responsiveness.

Our data showed that estrogen decreased *AIRE* expression (Figure 5B) in cultured mTECs, corroborating what have previously been shown (50). In addition, mRNA expression of *PLP* (an AIRE independent TSA) is significantly increased by estrogen (Figure 5C). More, we showed that the expression of the signaling molecule *IL-6* and *CCL21* is inhibited by estrogen while *TGF-β* mRNA expression remained unaffected. Altogether, these data suggest that estrogen activated in cultured human mTECs transduction pathways that regulated gene expression. Interleukin-1β induced no effect on *AIRE* expression (Figure 5B) while it inhibited mRNA expression of *PLP* (Figure 5C), highlighting that cultured mTECs display functional different transduction pathways (estrogen related network for AIRE, and inflammatory network for PLP) involved in the regulation of key tolerance molecules. More, IL-1β stimulated *IL-6* mRNA expression, and no effect was observed for *CCL21* and *TGF-β* mRNA expression (Figures 5D–F).

These data suggested that inflammatory and intrinsic factors modulate in primary cultured mTEC, expression of signaling molecules involved in T cell differentiation (IL-6 and CCL21) as well as in their own differentiation.

## Discussion

Here, we report that primary cultured of human thymic cells derived from human thymic explants, displayed mTEC features, remained functional to provide molecules for thymocyte education and maturation, and were able to respond to distinct specific signals from their environment. Even-though cTECs cannot be studied with our cultured cell model and this model is a short term culture model (7–8 days) that will promote the establishment of disease-specific thymic epithelial cell lines that could be used in long-term analysis.

Most studies have used mouse models to decipher tolerance and T-cell differentiation processes. So far, combined mechanic and enzymatic procedures, to extract or to obtain TECs, have been reported (34). These procedures, commonly used in mouse, require a significant amount of thymus biopsies. In addition, enzymatic dissociation is often associated with FACS-sorted or bead-sorted methods to purify murine and human TECs (59–61). These methods have helped to decipher with omics analyses (transcriptome, miRnome, exome, and proteome) a fixed picture of mTECs reflecting their crosstalk and interaction with other thymic cells. However, these methods are not followed by prolonged cell culture time due to the reduced extracted cell number and to the decreased cell viability (60). Therefore, we have optimized a technique that reduces the required amount of tissue and at the same time preserves cell viability and increases the number of extracted cells. In addition, human T-cells display specificities that require appropriate models that take into account human specificities. To this end, the establishment of human TEC cultures is a substantial step to address questions specific to the human T-cell development and differentiation. We observed that TEC cultures were functional, as molecular changes could be found after cytokine or hormone triggering. Thus, our culture method is not only useful for characterization of the epithelial cells *per se*, their morphology and surface characteristics, but also for their maturation process, secretion, and antigen presentation. This model will provide data, in humans and not mice, on the direct interactions between T-lymphocytes and thymic stromal cells, and could be the basis of the establishment of disease-specific thymic epithelial cell lines. Such method is the first step in the development of immortalized human mTEC lines (from control to disease-specific cell lines such as Myasthenia gravis or Omenn syndrome), models that will allow the understanding of the mechanisms underlining TEC function, thymic senescence, and aging as well as disease pathophysiological events.

### Human Viable Medullary TECs May Be Cultured From Human Thymic Explants

In our culture conditions, thymic-derived cells displayed specific mTEC markers (K5, K14, Claudin 4, and UEA-1) while cTEC markers were not detected (K8 and K18). Various groups have characterized a battery of markers such as cell membrane adhesion molecules (Claudin 4 and UEA-1), intracellular molecules (keratin 5/14), and transcription factors (Aire and FezF2) to identify mTECs. To this end, our TEC culture model provided cells that express these mTEC markers with a significant expression level. Still, whether these markers characterize thymic cells as mTECs (49), they do not define accurately the mTEC development and differentiation state (48, 62). For instance, most studies agreed that Claudin 3 and 4 are expressed by a subset of mTECs that co-expressed the UEA marker (49). But, the period of expression of such markers from mTEC progenitor through mature mTEC stage diverged between groups (48, 49, 62). Though some consensuses have emerged such as mTEC differentiation stages are characterized by differential MHC II, CD80 expression levels (63) while Aire expression is often associated with mature mTECs (30, 43, 63, 64).

Furthermore, mTECs development and differentiation depend upon activation of lymphotoxin β-receptor and TNF receptor (TNFR) (65). TNFR is activated by CD40L and RANKL, two cytokines produced in the thymus by lymphoid cells (45, 66, 67). These transduction pathways also regulate AIRE, FezF2, and PRDM1 expressions. As already observed and reported, in the primary cultured human mTECs, *AIRE* expression fluctuates during the culture process to reach its optimal expression around day 6–7 and then decline throughout the culture time (Figure S4 in Supplementary Material). Therefore, we can envisage that our cultured thymic explants provide signals to stimulate mTEC proliferation and differentiation through a certain period of time. Then, after 1 week, our culture method is devoid of the required lymphoid cells. A comparable observation was done for *AIRE* expression in another model of TEC culture (34) and in different murine TEC cultures (68, 69). In addition, gene decreased expression occurred quite often and is well documented for other proteins such as MHC II (70) or Fas (39), illustrating that primary cultured cells can lose throughout the culture time their ability to express specific factors, likely due to the depletion of specific cell types. In support to this hypothesis, the addition of RANKL, mainly produced by a CD4^+^ T cell subsets, restored the expression of *AIRE*, validating that the decline of expression for certain genes is probably due to the lack or decrease of a thymic specific microenvironment (1). FOXN1 is required to induce both cortical and medullary thymic epithelial cell differentiation (7, 20, 71, 72). Recently, O’Neill et al. (73) have suggested that “Post-AIRE expressing TECs, a potential distinct stage of terminal mTEC development, are sub-functional TEC resulting from downregulation of *Foxn1* and MHCII” (73). To confirm this hypothesis, our culture model may be an appropriate tool that will clarify human mTEC differentiation process.

### Human Derived mTECs Remain Keys Actor in T-Cell Maturation

Here, we demonstrated that cultured human primary mTECs do not lose their ability to provide signal molecules required for central tolerance basic mechanisms. A key hallmark of mTECs is their propensity to express TSAs to establish self-tolerance. AIRE, FEZF2 and PRDM1 are transcription factors identified to promote TSA expression in mTECs (13, 57, 63). In the thymus, where their expression is restricted to mTECs, they cooperate and do not displayed redundant roles as they modulate expression of different sets of TSAs in mTECs (13, 57, 74). Their essential roles, in negative selection or tolerance process, are highlighted in knockout mouse lines that develop autoimmune diseases with cell infiltrations in various peripheral tissues and autoantibody production (13, 57, 74).

In addition, mTECs secrete cytokines, chemokines, and growth factors (TGF-β, IL-6) molecules essential to maintain the T cell phenotype and to direct the differentiation and maturation in specific T cell subsets (65, 75, 76). In thymic medulla, TGF-β is mainly express by mTECs and in an autocrine manner regulates negatively the number of mTECs (77). TGF-β induces the expression of Foxp3, the Treg transcription factor in single positive CD4^+^ T cells (78). Therefore, TGF-β plays an important role on the differentiation and survival of thymic Treg cells (11) and reduces the escape or proliferation of autoreactive T cells from the medulla to the periphery (77). TGF-β may act in combination with IL-6 to stimulate the expression of retinoid-related orphan receptor, specific transcription factor of pro-inflammatory Th17 cells (79) in CD4^+^ T cells. IL-6, a pro-inflammatory cytokine, is produced mainly by thymic stroma cells (fibroblasts and TECs) (80) and is implicated in the differentiation of CD4^+^ T cell subsets and mediates B- and T-cell survival (81).

More, primary cultured human mTECs conserved their expression of chemokines, signal molecules mandatory to guide maturing T cells. Thus, *in vivo*, mTECs secrete several chemokines (CCL19, CCL21, CCL22, etc.) that attract T cells or DCs in the medulla during normal T cell maturation and migration throughout the thymus (82, 83). The migration of CD4^+^ T cells to the medulla is operated by an active chemotaxis process through the CCR7/CCR4, receptors expressed by CD4^+^ T cells (52). The sustained migration potentiates the interaction of differentiating thymocytes with thymic stromal cells, a process indispensable for their optimal differentiation and to perform the negative T cell selection (52, 84).

## Conclusion

We demonstrated that, similarly to *in vivo* situations, thymic-derived mTECs could respond with an appropriate answer to estrogen and inflammatory signals. TEC fate is a combination of crosstalks with T cells and the environment. Human mTECs in culture maintain their ability to express specific cytokines and chemokines, signal molecules mandatory to guide maturing T cells, to support the T cell phenotype and to direct the differentiation in particular T cell subsets. Besides, cultured human primary mTECs conserved their propensity to express TSA molecules required to enable the tolerance to self-molecules and to sensor their environment. These findings are in line with the report of Nazzal et al. (11) that demonstrate that human primary mTECs can promote the proliferation of newly generated CD25^+^ T cells from CD4^+^CD25^−^ T cells, protect Treg cells from cell death and preserve the phenotype and the function of Treg cells *in vitro*. As an interesting point of view, this model conserved the mTEC main features and functions. In the context of thymic related disease such as autoimmune myasthenia gravis, culturing mTECs is a good model to decipher the TEC physiopathological and functional disturbances underlining the chronic inflammation and the autoimmune reactions. Therefore, here we report a stable and robust model to culture human primary mTECs from the normal or pathological thymus. This model should yield valuable insight into the regulation of transduction pathway involved in thymic aging process as well as autoimmune pathological context.

## Ethics Statement

This study was carried out in accordance with the recommendations of by the local French ethics committee. The protocol was approved by the by the local ethics committee (CPP, Kremlin-Bicêtre, France: agreement No. 06-018; CCP Ile de France Paris 7, France agreement No. C09-36). Subjects have received an informed consent for the use of thymic fragments.

## Author Contributions

ND and JV performed the experiments, analyzed the data, and interpreted the results. FT provided helps to obtain tissues. RR provided human thymic tissues. AG and RLP provided helpful suggestions to design experiments. SB-A was involved in all aspects of the study. ND wrote the manuscript. All authors reviewed the manuscript.

## Conflict of Interest Statement

The authors declare that the research was conducted in the absence of any commercial or financial relationships that could be construed as a potential conflict of interest.

## Abbreviations

α-AChR, α-acetylcholine receptor; Aire, autoimmune regulator; BSA, bovine serum albumin; cTEC, cortical thymic epithelial cell; DC, dendritic cell; DHT, dihydrotestosterone; FBS, fetal bovine serum; Fezf2, Fez family zinc finger protein 2; GAD67, glutamic acid decarboxylase 67; HBSS, Hank’s balanced salt solution; IL-6, interleukin 6; IL-1β, interleukin 1β; K5, keratin 5; K8, keratin 8; K14, keratin 14; K18, keratin 18; Lt-β, lymphotoxin-β; mTEC, medulla thymic epithelial cell; NKT, natural killer T cell; PBS, phosphate-buffered saline; PLP, myelin proteolipid protein; Prdm1, PR domain zinc finger protein 1; Rankl, receptor activator of nuclear factor kappa-B ligand; TGF-β, tumor growth factor-β; TG, thyroglobulin; Treg, regulatory T cell; TNFR, TNF receptor; TSA, tissue-specific antigen; UEA, *Ulex europaeus* agglutinin-1.
