# Supplementary material for: Cultured Human Thymic-Derived Cells Display Medullary Thymic Epithelial Cell Phenotype and Functionality
Source: Front Immunol. 2018 Jul 23;9:1663. doi: 10.3389/fimmu.2018.01663 (PMC6064927; doi:10.3389/fimmu.2018.01663)
Supplement: Supplementary file 1 [file data_sheet_1.PDF]

CULTURED HUMAN THYMIC-DERIVED CELLS DISPLAY MEDULLARY THYMIC EPITHELIAL CELL PHENOTYPE AND FUNCTIONALITY

José Villegas [1], Angeline Gradolatto [1], Frédérique Truffault [1], Régine Roussin [2], Sonia Berrih-Aknin[1], Rozen Le Panse [1]and Nadine Dragin [1, 3*],

Sorbonne University, INSERM, AIM, Center of research in Myology, UMRS974, Paris, France

[1] Sorbonne University, INSERM, AIM, Center of research in Myology, UMRS974, Paris, France.

[2] Hospital Marie Lannelongue, Le Plessis-Robinson, France

[3] Inovarion, Paris, France

[*] Corresponding author

Correspondence and Requests for materials should be addressed to:

Dr Nadine Dragin, Sorbonne University - INSERM, AIM, Center of Research in Myology, UMRS974, 105 Bd de l'hôpital, 75013 Paris, France, Tel: 00 33 (0)1 40 77 81 27, Fax: 00 33 (0)1 40 77 81 29; nadine.dragin@inovarion.com

Keywords: Thymic epithelial cells, primary cell culture method, keratins, cytokines, chemokines, tissue-specific antigens.

Supplemental table 1: List of antibodies used in the study

Antigen	Clone	Manufacturer
CLAUDIN 4	382321	R&D
COLLAGEN III	Clone III-53	ICN/biomed
KERATIN 14	AF 64	COVANCE
KERATIN 5	AF 138	COVANCE
MNF-116	MO 821	Dako
UEA		Vector laboratories

Supplemental table 2: List of primer used in the study

GENE NAME	FORWARD PRIMER	REVERSE PRIMER
α-AChR	AAGCTACTGTGAGATCATCGTCAC	TGACGAAGTGGTAGGTGATGTCCA
AIRE	ATGACACTGCCAGTCACGAG	AGGAGGTGTCCTTCTCAGCA
CCL19	GGTGCCTGCTGTAGTGTTCA	GGTCCTTCCTTCTGGTCCTC
CCL21	CAAGCTTAGGCTGCTCCATC	TCAGTCCTCTTGCAGCCTTT
COLLAGEN III	TACGGCAATCCTGAACTTCC	GTGTGTTTCGTGCAACCATC
FEZf2	CGGCTCCCTATCCCCATAAG	TGAGCGTTAAACACCTTGCC
GAD 67	CACAAGGTGGCTCCAAAAAT	TTACAGATCCTGGCCCAGTC
GAPDH	GCTGAGTACGTCGTGGAGTC	GATGATGTTCTGGAGAGCCC
IL-6	TACCCCCAGGAGAAGATTCC	GCCATCTTTGGAAGGTTCAG
KERATIN 5	GGTTGATGCACTGATGGATG	TCCTCATACTGGGCCTTGAC
KERATIN 8	TGAGGTCAAGGCACAGTACG	TGATGTTCCGGTTCATCTCA
KERATIN 14	TTCTGAACGAGATGCGTGAC	GCAGCTCAATCTCCAGGTTC
PLP	CTTCAACACCTGGACCACCT	AGCATTCCATGGGAGAACAC
PRDM1	AAGCAACTGGATGCGCTATGT	GGGATGGGCTTAATGGTGTAGAA
THYROGLOBULIN	CCTGCTGGCTCCACCTTG	CCTTGTTCTGAGCCTCCC
TGF-β	GGGACTATCCACCTGCAAGA	CCTCCTTGGCGTAGTAGTCG
