# Supplementary material for: Cultured Human Thymic-Derived Cells Display Medullary Thymic Epithelial Cell Phenotype and Functionality
Source: Front Immunol. 2018 Jul 23;9:1663. doi: 10.3389/fimmu.2018.01663 (PMC6064927; doi:10.3389/fimmu.2018.01663)
Supplement: Supplementary file 2 [file data_sheet_2.PDF]

CULTURED HUMAN THYMIC-DERIVED CELLS DISPLAY MEDULLARY THYMIC EPITHELIAL CELL PHENOTYPE AND FUNCTIONALITY

José Villegas [1], Angeline Gradolatto [1], Frédérique Truffault [1], Régine Roussin [2], Sonia Berrih-Aknin[1], Rozen Le Panse [1]and Nadine Dragin [1, 3*],

Sorbonne University, INSERM, AIM, Center of research in Myology, UMRS974, Paris, France

[1] Sorbonne University, INSERM, AIM, Center of research in Myology, UMRS974, Paris, France.

[2] Hospital Marie Lannelongue, Le Plessis-Robinson, France

[3] Inovarion, Paris, France

[*] Corresponding author

Correspondence and Requests for materials should be addressed to:

Dr Nadine Dragin, Sorbonne University - INSERM, AIM, Center of Research in Myology, UMRS974, 105 Bd de l'hôpital, 75013 Paris, France, Tel: 00 33 (0)1 40 77 81 27, Fax: 00 33 (0)1 40 77 81 29; nadine.dragin@inovarion.com

Keywords: Thymic epithelial cells, primary cell culture method, keratins, cytokines, chemokines, tissue-specific antigens.

Supplemental Figure 1:

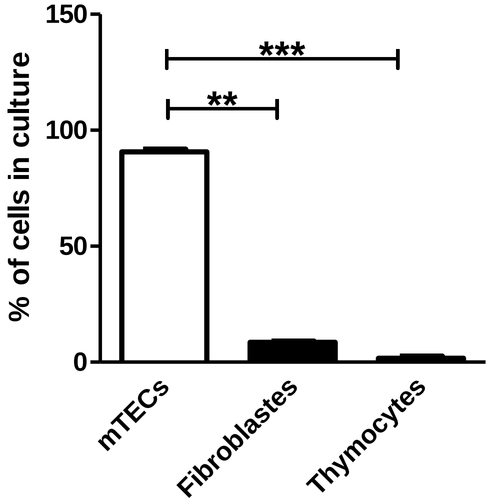

Primary cultured human TECs were trysinized at day 7. Cells were stained with a rabbit anti-CD45 antibody, fixed, permeabilized to be labeled with anti-Collagen III and anti-MNF116 antibodies. Labelled cells were analyzed on the FACSVerse apparatus using the Facs suite software (Becton Dickinson, Le Pont de Claix, France).

Graph bars represent the combined results of different primary cultured human TECs obtained with at least four different human biopsies. P values were obtained using the non-parametric Mann-Whitney test. Asterisks indicate significant differences (***$p<0.003$; ***$p<0.0008$*).

Supplemental Figure 2:

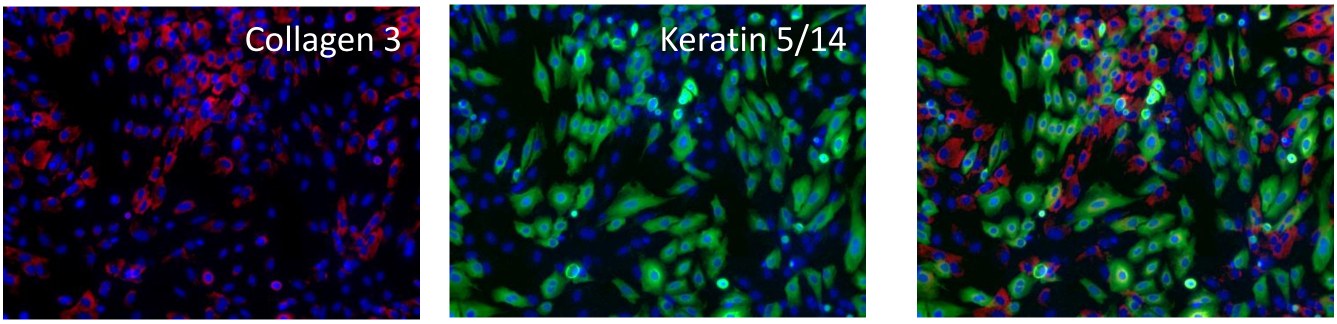

Supplemental figure 2: Pictures of a primary cultured human TECs (day 7) co-labeled with an anti-Collagen III antibody (red), anti-Keratin 5 and 14 antibodies (green), and DAPI (blue). While the large majority of cells are epithelial (Keratin 5/14-positive), some cells are collagen III-positive, reflecting the fibroblast contamination.

Supplemental Figure 3:

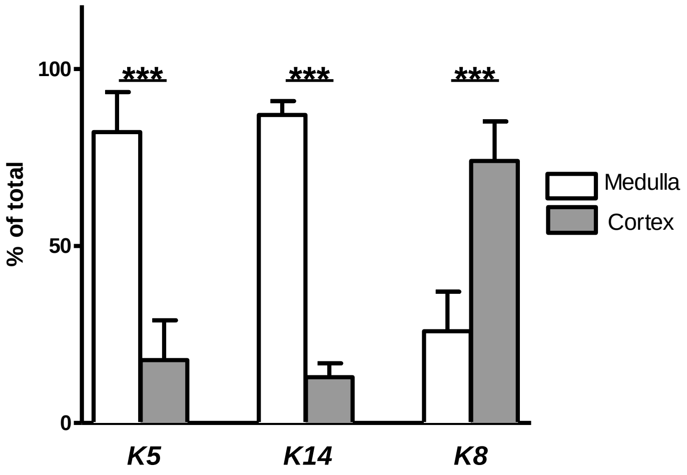

Frozen human thymic sections were stained with eosin-hematoxylin. Thymic medulla and cortical areas were micro-dissected from eight different human thymic sections. Total mRNA were extracted with the RNA extraction Qiagen Kit. Gene mRNA expressions were analyzed by real-time PCR and normalized to *28S*. Percentage of gene expression per thymic area represents the following ratio :(M/(M+C))*100 or (C/(M+C))*100 respectively for the medulla (M) and the cortex (C).
P values were obtained using the non-parametric Mann-Whitney test. Asterisks indicate significant differences (*** *p<0.001*).

Supplemental Figure 4:

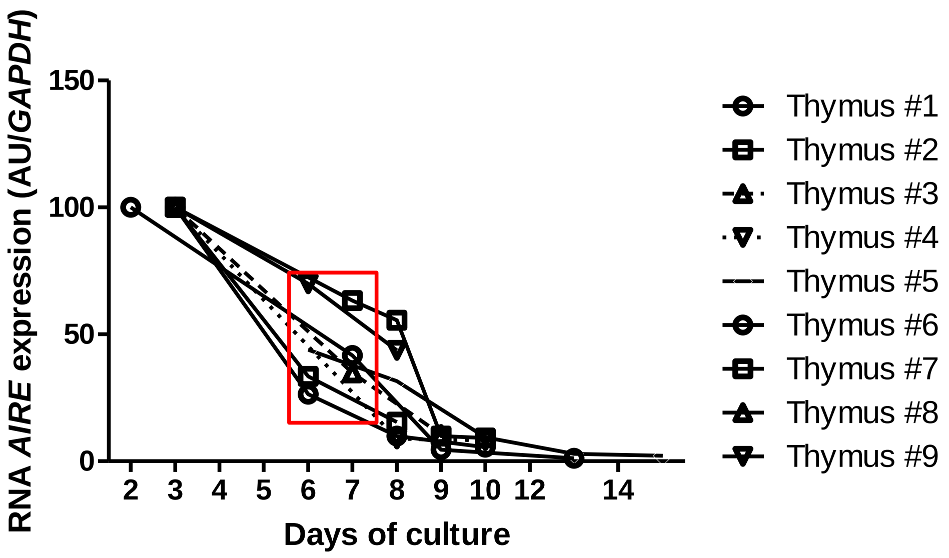

Supplemental Figure 4: mRNA expression of *AIRE* in primary thymic epithelial cells throughout the culture time. N=9 primary cultured human TECs obtained from 9 different thymi. Total mRNA were extracted with the RNA extraction Qiagen Kit. Gene mRNA expressions were analyzed by real-time PCR and normalized to GAPDH.
